# Tracing the Geographical Origin of Thai Hom Mali Rice in Three Contiguous Provinces of Thailand Using Stable Isotopic and Elemental Markers Combined with Multivariate Analysis

**DOI:** 10.3390/foods10102349

**Published:** 2021-10-01

**Authors:** Supalak Kongsri, Phitchan Sricharoen, Nunticha Limchoowong, Chunyapuk Kukusamude

**Affiliations:** 1 Nuclear Technology Research and Development Center (NTRDC), Thailand Institute of Nuclear Technology (Public Organization), 9/9 Moo 7, Saimoon, Ongkharak, Nakhon Nayok 26120, Thailand; supalak@tint.or.th (S.K.); phitchan@tint.or.th (P.S.); 2 Department of Chemistry, Faculty of Science, Srinakharinwirot University, Sukhumvit 23, Wattana, Bangkok 10110, Thailand; nuntichoo@gmail.com

**Keywords:** traceability, geographical origin, Thai Hom Mali rice, ICP-MS, IRMS, food authenticity, multivariate analysis, discriminant analysis

## Abstract

Rice is a staple food for more than half of the world’s population. The discrimination of geographical origin of rice has emerged as an important issue to prevent mislabeling and adulteration problems and ensure food quality. Here, the discrimination of Thai Hom Mali rice (THMR), registered as a European Protected Geographical Indication (PGI), was demonstrated. Elemental compositions (Mn, Rb, Co, and Mo) and stable isotope (δ^18^O) in the rice were analyzed using inductively coupled plasma mass spectrometry (ICP-MS) and elemental analyzer isotope ratio mass spectrometry (EA-IRMS), respectively. The recoveries and precisions of all elements were greater than 98% and lower than 9%, respectively. The analytical precision (±standard deviation) was below ±0.2‰ for δ^18^O measurement. Mean of Mn, Rb, Co, Mo, and δ^18^O levels was 14.0 mg kg^−1^, 5.39 mg kg^−1^, 0.049 mg kg^−1^, 0.47 mg kg^−1^, and 25.22‰, respectively. Only five valuable markers combined with radar plots and multivariate analysis, linear discriminant analysis (LDA) could distinguish THMR cultivated from three contiguous provinces with correct classification and cross-validation of 96.4% and 92.9%, respectively. These results offer valuable insight for the sustainable management and regulation of improper labeling regarding geographical origin of rice in Thailand and other countries.

## 1. Introduction

Rice is a major component of calories [1] and carbohydrates [2] for human beings. It is also a main source of vitamins and minerals [3]. Therefore, rice is an important staple food for more than 50% of the world’s population [2,3,4] especially American, European, and Asian [5]. About 90% of rice is produced in Asia. The top six largest rice-producing countries are China, India, Indonesia, Bangladesh, Vietnam, and Thailand [6]. Thailand exported 5.72 million tonnes of rice in 2020 with an export value of 115.9 billion baht (3.7 billion US dollars).

Thai Hom Mali rice (THMR) is also known worldwide and was recorded under the European Protected Geographical Indication (PGI) scheme in 2013 [7]. THMR cultivated in the center of Northeastern Thailand is annually grown and can be harvested only once a year during the months of October to November [8]. The climate condition combined with minerals in soil cause tension to the rice making it produce an aromatic substance, 2-acetyl-1-pyrroline [9]. It has a long grain with moist, silky smooth, soft texture, and sweet-scented aroma after cooking. THMR cultivated in the Thung Kula Rong-Hai area is recognized as premium quality rice. Therefore, THMR has been adulterated with lower-grade rice from other areas in order to increase profits. Furthermore, mislabeled rice is at higher risk of food safety issues such as harmful unspecified rice additives and contaminants. Food fraud has become enlarged to a global scale because of increasing demand in food [10]. Thus, it is important to protect rice consumers from commercial fraud. These concerns directly impact global consumers, exporters, and countries. In this regard, authenticity and traceability measurements have become important issues in food testing.

Genetic and environmental factors (weather), crop management, season, and soil composition affect the elemental and isotopic characteristics in rice [11,12]. Moreover, the oxygen isotope in rice originates from local groundwater, meltwater, and precipitation [13]. Enriched δ^18^O in rice is expected in growing regions with relatively low humidity and a high transpiration rate [12]. However, the topography and climate conditions must be considered. Contaminated soil and water in rice manufacturing are the main elemental sources that can be taken up by plant roots. Therefore, rice has unique elemental profiles reflecting the geochemistry of the soil origin [14]. The determination of rice’s geographical origin can be performed using a combination of chemometric approaches with several reliable analytical techniques such as instrumental neutron activation analysis (INAA) [2,15,16], isotope ratio mass spectrometry (IRMS) [13,17,18,19,20], inductively coupled plasma optical emission spectrometry (ICP-OES) [21,22,23,24,25], inductively coupled plasma mass spectrometry (ICP-MS) [1,23,24,26,27,28,29], LA-ICP-MS [30], and high resolution inductively coupled plasma mass spectrometry (HR-ICP-MS) [5,25,31]. However, the combination of multi-elemental and isotopic techniques with chemometric approaches has been developed for tracing the geographical origin of foodstuffs [32]. The applications of isotopic and elemental approaches have been also reported for the discrimination of the geographical origin of rice [2,12,13,33,34,35]. Nine variables (δ^13^C, δ^18^O, B, Mg, Se, Rb, Gd, Ho, and W) combined with canonical discriminant analysis were used to determine the geographical origin of rice from India, Pakistan, Europe (France, Italy, and Spain), and USA [12]. Stable isotope compositions δ^13^C, δ^15^N, δ^18^O, C, and N with radar plots were used to discriminate the polished rice from Australia, Japan, and USA [13]. Geographical authentication for rice collected from six Asian countries was discriminated by δ^13^C, δ^15^N, δ^18^O, and δ^34^S and 25 elements (Mg, K, Ca, B, Mn, Fe, Cu, Zn, Mo, Be, Na, Al, Ti, V, Cr, Co, Ni, As, Se, Sr, Ag, Cd, Ba, Tl, and Pb) combined with principal component analysis (PCA) and orthogonal projection to latent structure-discriminant analysis [33]. Seven stable isotope ratios (δ^13^C, δ^15^N, δ^2^H, δ^18^O, ^87/86^Sr, ^207/206^Pb, and ^208/207^Pb) and 25 elements (Li, Be, V, Cr, Co, Ni, Cu, Rb, Sr, Mo, Ag, Cd, Sn, Pb, Al, Ti, Mn, Fe, Zn, Sb, Ba, Bi, Na, Mg, and Ca) combined with PCA and linear discriminant analysis (LDA) were established to differentiate polished rice from different growing regions in China with rice imported from Southeast Asia (Thailand and Malaysia) [35].

Thus, the objective of the study was to investigate the isotopic and elemental markers for tracing the geographical origins of THMR cultivated in three contiguous provinces in Thailand. The three provinces include Yasothon province (YP), Roi Et province (RP), and Surin province (SP). Isotopic and elemental markers in THMR were analyzed using ICP-MS and EA-IRMS. In this study, the obtained results from ICP-MS and EA-IRMS were analyzed by radar plots and multivariate analysis, LDA. Discrimination power for candidate markers was also evaluated. The work hereafter demonstrates the feasibility study of using elemental and isotopic compositions to determine the geographical origins of THMR cultivated in three contiguous provinces in Thailand, which is one of the most predominant rice consumption, exporter, and production country. Hence, these results could be potentially used for sustainable management and regulation of proper labeling in the geographical origin of rice in Thailand and other countries. 

## 2. Materials and Methods

### 2.1. Sample Preparation of THMR

**Figure 1:**
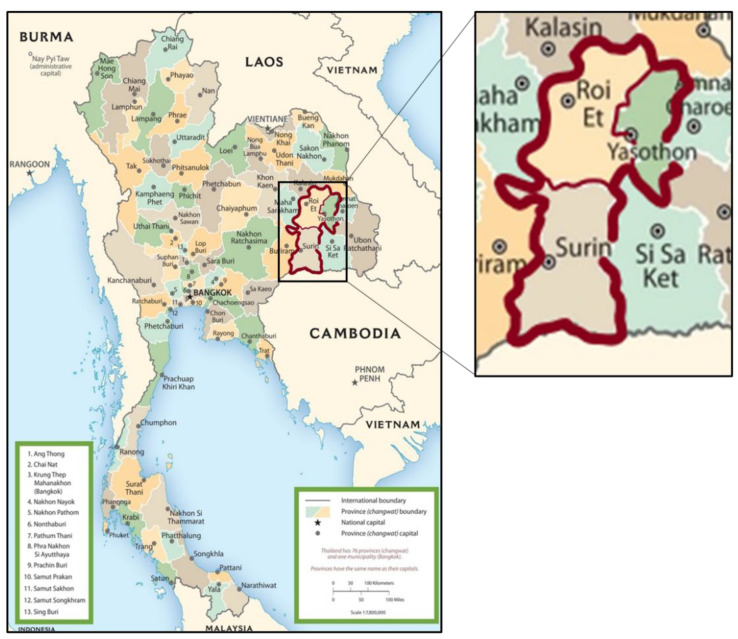
Sampling areas of three contiguous provinces in Thailand for Thai Hom Mali rice (THMR) samples. The right figure is the enlargement of Yasothon province (YP), Roi Et province (RP), and Surin province (SP).

Twenty-eight THMR samples were cultivated from three provinces including YP (n = 7), RP (n = 16), and SP (n = 5). A map of the sampling provinces is shown in Figure 1. Before the polished rice samples were pulverized by Perten Laboratory mill 3100 (Perten Instruments, Huddinge, Sweden), all rice samples were air-dried. The fine powder of the rice sample was then further dried in oven at 60 °C until dry. All analytical techniques were performed at Thailand Institute of Nuclear Technology (Public Organization).

### 2.2. Chemicals and Reference Materials

Ultrapur HNO_3_ and suprapur H_2_O_2_ were obtained from Merck (Darmstadt, Germany). Calibration standards were diluted in 2% (*v*/*v*) HNO_3_. Calibration standard, internal standard solution, tuning solution (Agilent Technologies, Santa Monica, CA, USA) and rice flour SRM 1568b (NIST, Gaithersburg, MD, USA) were used for ICP-MS analysis. EA-IRMS measurement was performed using international reference materials including USGS35 (USGS, Reston, VA, USA), IAEA-CO-8, IAEA-601, and IAEA-602 (IAEA, Vienna, Austria) with δ^18^O values of +57.5 ± 0.3‰, −22.7 ± 0.2‰, +23.14 ± 0.19‰, and +71.28 ± 0.36‰, respectively.

### 2.3. ICP-MS

THMR was digested by microwave digestion prior to Agilent 7900 ICP-MS analysis. Fine powder of rice (800 mg) was weighted into a vessel. Concentrated HNO_3_ (9 mL) and H_2_O_2_ (1 mL) were added. Then, the closed vessel was digested using MARS 6 (CEM). Microwave digestion was set at 160 °C and held for half an hour. Afterward, the solution was dried and redissolved in 2% (*v*/*v*) HNO_3_ (10 mL). Conditions of ICP-MS are described in Supplementary Table S1.

### 2.4. Stable Oxygen (O) Isotope by EA-IRMS in Pyrolyzer Mode

The stable O isotope in THMR was determined using an Elementar Vario PYRO cube elemental analyzer interfaced to an Isoprime 100 IRMS system (EA-IRMS, Isoprime Ltd., Manchester, UK). The stable isotope of O was determined in terms of isotope ratios (δ^18^O). The powdered rice samples kept in the desiccator were accurately weighed into 4 × 4 × 11 mm silver boats (0.3 mg). The samples in the enclosed capsules were thermally decomposed to CO gas in a glassy carbon reactor filled with glassy carbon granules, carbon black, and graphite felt at 1450 °C. The carrier gas flow transfers the gaseous pyrolysis products into the adsorption tube where acid and alkaline pyrolysis products were absorbed chemically. The CO gas was isolated from natural gaseous component (N_2_ gas) by an adsorption column and then the CO gas was subjected to the IRMS system. The values (‰) were denoted in delta against the international reference standard (Vienna Standard Mean Ocean Water, VSMOW) for δ^18^O, according to the following general Equation (1) [2,33]:

(1) $$\delta^{18}O\;\left(‰\right)=(\left(R_{sample}-R_{standard}\right)/R_{standard})\times 1000$$

where *R_sample_* and *R_standard_* are the stable isotope ratio (^18^O/^16^O) of the sample and the international reference standard (VSMOW), respectively. Two-point linearity correction was performed for the measurement. In this study, the analytical precision (±standard deviation) for δ^18^O measurements was below ±0.2‰ on the basis of the reference materials.

### 2.5. Multivariate Analysis

The multivariate analysis of the results obtained by ICP-MS and EA-IRMS was analyzed using IBM SPSS statistics version 23. Significance was detected at the level of *p*-value less than 0.05 using ANOVA and Kruskal–Wallis test. Normally distributed data were treated with ANOVA using Tukey HSD and Games–Howell post hoc test, while Kruskal–Wallis was used for non-normal distribution with Dun–Bonferroni post hoc.

To determine the factors for differentiating the geographical origins of THMR, LDA was conducted in this study. The transformed data were subsequently subjected to LDA. LDA was combined with elemental compositions (Mn, Rb, Co, and Mo) and stable isotope (δ^18^O) obtained from 3 contiguous provinces.

## 3. Results and Discussion

### 3.1. Stable Isotope and Selected Elemental Analyses in THMR

#### 3.1.1. δ^18^O Analysis

The ^18^O value was used as an effective marker for the determination of geographical origin because of different physical, biological, and chemical processes from different environments leading to isotopic fractionation. The δ^18^O values in agricultural products are correlated to geological structures, irrigation, geography and climate, distance from coast, precipitation, etc. [12,33]. The δ^18^O values were determined using EA-IRMS set in pyrolyzer mode. The mean δ^18^O value (25.22‰) in the studied THMR samples was slightly lower than that in Indica rice (+25.8‰) [4], while it was 1.14-fold higher than that in Indica rice (+22.15‰) [33]. However, the mean δ^18^O value was similar to those in polished rice and jasmine rice in the previous studies [2,20]. The order of δ^18^O values of rice in several countries was Spain (+28.4‰) > USA (+26.3‰) [12] > Thailand, in this study (+25.22‰) > France (+25.1‰) > Italy (+24.5‰) [12] ≥ Vietnam (+24.5‰) [20] > Pakistan (+23.3‰) [12] > USA (+22.9‰) [13] > India (+22.1‰) [12] > Japan (+21.3‰) [20] > Australia (+20.3‰) [13] > Japan (+19.7‰) [13]. In this study, the geographical δ^18^O variations in THMR in YP, RP, and SP are summarized in Table 1. δ^18^O in THMR grown in YP, RP, and SP was in the ranges of +24.69‰ to +26.00‰, +24.32‰ to +26.67‰, and +23.65‰ to +24.71‰, respectively, with relative standard deviation (RSD) of 0.018, 0.03, and 0.018%, correspondingly.

**Figure 2:**
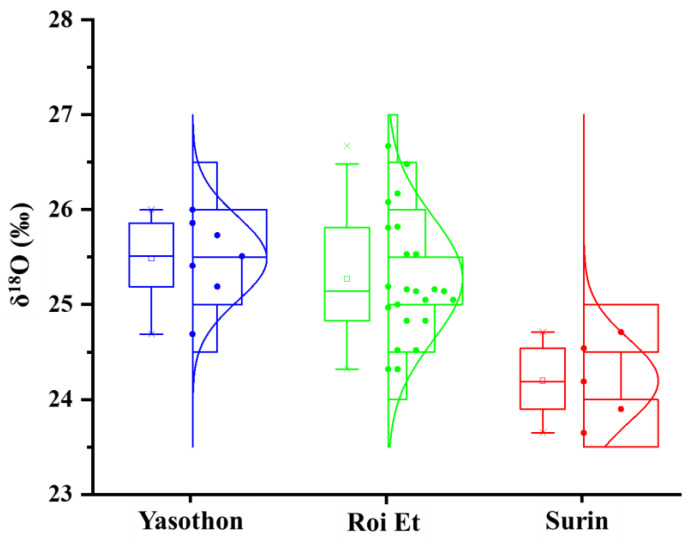
Box-and-whisker plots showing the variation of stable isotope, δ^18^O values in THMR collected from three contiguous provinces in Thailand covering YP (n = 7), RP (n = 16), and SP (n = 5), respectively.

Figure 2 shows the variations of δ^18^O values in THMR of interest according to the geographical origins. Mean δ^18^O values in THMR cultivated in YP, RP, and SP were +25.48‰, +25.42‰, and +24.20‰, respectively. The mean δ^18^O values in THMR varied significantly with the province of origin (*p* = 0.001). The result of the Tukey HSD post-hoc test was explained in Table 1. Different letters (a, b) show significant differences in the mean of δ^18^O values of each province examined in this study based on a Tukey HSD test at 0.05 probability. The mean δ^18^O values in THMR between SP and YP and SP and RP were significantly different with a *p*-value of 0.003 and 0.002, respectively, probably due to different rainfall, precipitation, and temperature over each region. This indicates that δ^18^O was a good indicator for identification of geographical origin of THMR cultivated from SP. However, using only one parameter, δ^18^O showed weak discriminatory power for identification of geographical origins of THMR (data not shown). Therefore, some elements were studied in order to increase discriminatory power to discriminate of geographical origin of THMR grown in three contiguous provinces.

#### 3.1.2. Elemental Analysis

Validation of the method followed the Eurachem Guide [36]. Method validation for the analysis of Mn, Co, Rb, and Mo in Thai Hom Mali rice by ICP-MS are illustrated in Supplementary Table S2. The evaluation of linearity was done using four to six levels of standard solution. Good linearity for the selected metals was greater than 0.9990. Limits of quantification (LOQs) and limits of detection (LODs) ranged from 0.83 to 12.4 μg kg^−1^ and 0.29 to 11.8 μg kg^−1^, respectively. Accuracy of the method was assessed by evaluating the matrix-matched certified reference material (SRM 1568b). The recovery of the elements was higher than 98%. Precisions were <9%.

The use of multi-element analysis for the determination of the geographical origin of rice has been studied [2,12,23,25,37]. Regarding the authentication of geographical origin of rice, mineral or elemental profiling has been used. The mineral occurrence in food correlates with the soil type and environmental growing conditions.

**Figure 3:**
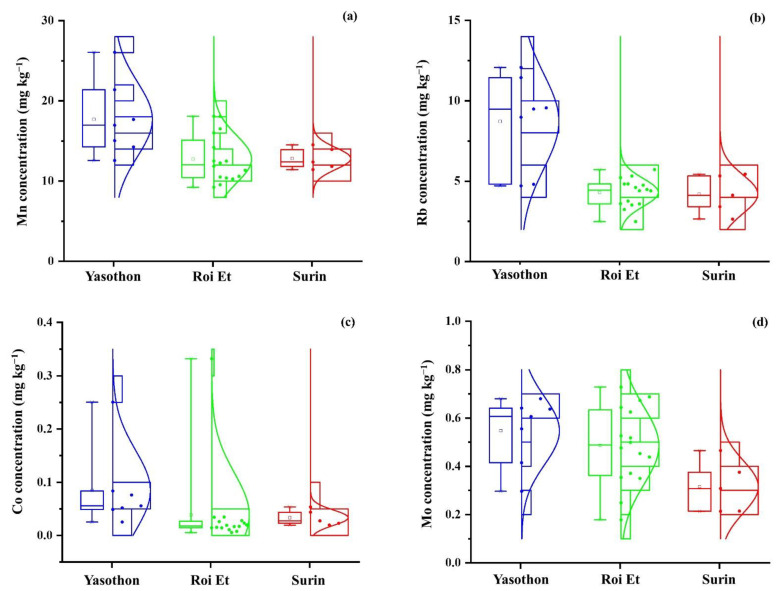
Box-and-whisker plots showing the variation of (**a**) Mn, (**b**) Rb, (**c**) Co, and (**d**) Mo concentrations in THMR collected in three contiguous provinces in Thailand covering YP (n = 7), RP (n = 16), and SP (n = 5), respectively.

In this study, metals including Mn, Rb, Co, and Mo in 28 samples of THMR collected in three different provinces were determined using ICP-MS. Concentrations of four elements in THMR by ICP-MS are illustrated by box-and-whisker plots in Figure 3. Mn (Figure 3a), Rb (Figure 3b), Co (Figure 3c), and Mo (Figure 3d) concentrations in THMR were found in the ranges of 9.23–26.05, 2.49–12.07, 0.006–0.33, and 0.18–0.73 mg kg^−1^, respectively. Mean ± S.D. of Mn, Rb, Co, and Mo concentrations in THMR samples was 14.00 ± 3.8, 5.39 ± 2.5, 0.049 ± 0.07, and 0.47 ± 0.2 mg kg^−1^, correspondingly. The mean Mn level in this study was about 1.6–4 folds higher than those in Thai Indica rice, Thai jasmine white rice, and white rice samples from northern Thailand published in the previous studies [5,30,33]. The mean concentration of Rb was around 1.2–1.8 times higher than those in Thai jasmine white rice and white rice samples in the previous studies [5,30]. In this study, the mean Co level in THMR was 1.3–1.7 times higher than those levels, while the mean Mo level was almost similar to those levels in the previous studies [5,30]. The order of Mn levels in rice from several countries was Brazil (60 mg kg^−1^) [22] > Philippines (23.83 mg kg^−1^) > Cambodia (18.07 mg kg^−1^) [33] > Malaysia (16.61 mg kg^−1^) [16] > China (16.55 mg kg^−1^) [33] > Thailand, in this study (14.00 mg kg^−1^) ≥ Japan (14 mg kg^−1^) > Spain (13 mg kg^−1^) > India (11 mg kg^−1^) [22] > Korea (10.70 mg kg^−1^) [33] > Pakistan (9.10 mg kg^−1^) > Italy (7.75 mg kg^−1^) > India (6.99 mg kg^−1^) > Japan (6.71 mg kg^−1^) > France (5.41 mg kg^−1^). Rb levels in rice from several countries were in the order of Malaysia (13.06 mg kg^−1^) [16] > Thailand, in this study (5.39 mg kg^−1^) > France (3.12 mg kg^−1^) > Pakistan (2.46 mg kg^−1^) > USA (1.99 mg kg^−1^) > India (1.80 mg kg^−1^) [12] > India (1.67 mg kg^−1^) > Pakistan (1.23 mg kg^−1^) [5] > Italy (1.22 mg kg^−1^) > Spain (0.92 mg kg^−1^) [12] > France (0.67 mg kg^−1^) > Italy (0.41 mg kg^−1^) > Japan (0.18 mg kg^−1^) [5]. The order of Co levels in rice from several countries was Thailand, in this study (0.049 mg kg^−1^) > Cambodia (0.037 mg kg^−1^) > Philippines (0.032 mg kg^−1^) [33] > India (0.007 mg kg^−1^) > Italy (0.006 mg kg^−1^) ≥ Pakistan (0.006 mg kg^−1^) [5] ≥ Japan (0.006 mg kg^−1^) [33] > USA (0.0044 mg kg^−1^) [31] > Japan (0.004 mg kg^−1^) [5] ≥ China (0.004 mg kg^−1^) > Korea (0.003 mg kg^−1^) [33] > France (0.002 mg kg^−1^) [5]. Mo levels in rice from several countries were in the order of India (1.38 mg kg^−1^) > Pakistan (1.22 mg kg^−1^) > Italy (0.98 mg kg^−1^) > Japan (0.79 mg kg^−1^) [5] > India (0.7 mg kg^−1^) [22] > Japan (0.632 mg kg^−1^) > Philippines (0.594 mg kg^−1^) [33] > France (0.5 mg kg^−1^) [5] > Thailand, in this study (0.47 mg kg^−1^) > Brazil (0.4 mg kg^−1^) [22] > China (0.3 mg kg^−1^) > Cambodia (0.27 mg kg^−1^) > Korea (0.232 mg kg^−1^) [33] > Japan (0.2 mg kg^−1^) [22].

Mn concentrations in THMR cultivated in YP, RP, and SP were in ranges of 12.57–26.05 mg kg^−1^, 9.23–18.08 mg kg^−1^, and 11.43–14.53 mg kg^−1^, respectively. The mean Mn concentrations were 17.71, 12.74, and 12.82 mg kg^−1^ in THMR cultivated in YP, RP, and SP, respectively, as summarized in Table 1. Significant differences in Mn concentrations in THMR cultivated in three different provinces using Kruskal–Wallis test were obtained with the *p*-value of 0.017_._ Based on the Dun–Bonferroni, there were statistically significant differences in Mn concentrations in THMR cultivated in YP and RP. There was no significant difference in Mn concentration found in THMR between YP and SP (*p* = 0.157) and between RP and SP (*p* = 1.000).

Rb concentrations in THMR cultivated in YP, RP, and SP were in ranges of 4.71–12.07 mg kg^−1^, 2.49–5.72 mg kg^−1^, and 2.64–5.44 mg kg^−1^, respectively. In Table 1, mean Rb concentrations were 8.72, 4.30, 4.19 mg kg^−1^ in THMR cultivated in YP, RP, and SP, respectively. Significant differences (*p* < 0.001) in Rb concentrations in THMR cultivated in three different provinces were observed using ANOVA. The Games–Howell post-hoc test showed that mean Rb concentrations in THMR cultivated from YP were significantly different from other origins (RP and SP). There was no significant difference in Rb concentration obtained in THMR between RP and SP at 95% confidence interval.

As summarized in Table 1, the mean concentrations of Co in THMR cultivated in YP, RP, and SP were 0.085, 0.039, and 0.033 mg kg^−1^, respectively. Co concentrations in THMR cultivated in YP, RP, and SP were in ranges of 0.025–0.25 mg kg^−1^, 0.0055–0.33 mg kg^−1^, and 0.020–0.054 mg kg^−1^, respectively. Significant differences in Co concentrations in THMR cultivated from 3 different provinces using Kruskal–Wallis test were obtained with the *p*-value of 0.004_._ Based on the Dun–Bonferroni, there were statistically significant differences in Co concentrations in THMR cultivated in YP and RP. There was no significant difference in Co concentration in THMR between YP and SP (*p* = 0.529) and between RP and SP (*p* = 0.501).

Concentrations of Mo in THMR cultivated in YP, RP, and SP were in ranges of 0.30–0.68 mg kg^−1^, 0.18–0.73 mg kg^−1^, and 0.21–0.47 mg kg^−1^, respectively. As summarized in Table 1, mean Mo concentrations were 0.55, 0.49, 0.32 mg kg^−1^ in THMR cultivated in YP, RP, and SP, respectively. Significant differences in Mo concentrations in THMR cultivated in three different provinces were obtained with the *p*-value of 0.038 using ANOVA. Based on the Tukey HSD, there were significant differences in Mo concentrations in THMR cultivated in YP and SP with *p*-value of 0.034. There was no significant difference in Mo concentration in THMR between YP and RP (*p* = 0.636) and between RP and SP (*p* = 0.084) at 95% confidence interval.

### 3.2. Radar Plot Analysis

**Figure 4:**
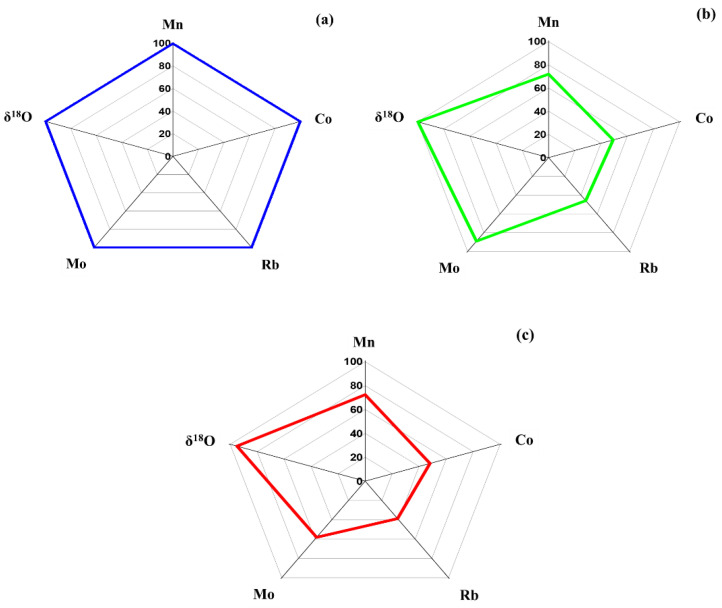
Radar plots summarizing the difference as relative concentration of the studied variables (Mn, Co, Rb, Mo, and δ^18^O) in THMR from (**a**) YP, (**b**) RP, and (**c**) SP.

A simple and rapid way to distinguish the geographical origin of rice samples based on elemental and isotopic composition has been used [2,5,13]. To simplify the comparison and discrimination according the geographical variation, the significant difference parameters (Mn, Rb, Co, Mo, and δ^18^O) were used for radar plot. The radar plots of Mn, Rb, Co, Mo, and δ^18^O variables summarized as relative concentration of five variables are shown in Figure 4.

Figure 4 shows the isotopic and elemental distribution patterns of THMR in 3 different provinces of the geographical origins including YP (Figure 4a), RP (Figure 4b), and SP (Figure 4c). Figure 4 shows the unique patterns of THMR cultivated in three contiguous provinces of the geographical origins. This revealed the possibility to identify the geographical origins of THMR. In order to ensure the geographical origin of THMR, LDA was further studied.

### 3.3. LDA

Isotopic and elemental compositions (δ^18^O, Mn, Rb, Co, and Mo) in THMR cultivated from 3 geographical origins were performed using ICP-MS and EA-IRMS. LDA is a statistical approach that maximizes between-group variance and minimizes within-group variance [38]. Discrimination analysis was applied to classify groups and also assign the samples to the groups. LDA of THMR from three provinces was undertaken to classify province of origin. Using only elemental compositions (four metal variables), an LDA model was initially built, but it’s not enough to discriminate the origins. THMR cultivated from SP could be distinguished from other provinces with a discrimination accuracy of 100%. However, for other provinces (YP and RP), the discrimination accuracy was 85.7 and 75%, respectively. Overall, the geographical discrimination accuracy was 82.1% using only the elemental compositions (Table 2).

Finally, using combining stable isotope and elemental composition data (five variables), most provinces were well classified as 85.7% (YP), 100.0% (RP), and 100.0% (SP), correspondingly. The overall discrimination accuracy increased to 96.4%.

**Figure 5:**
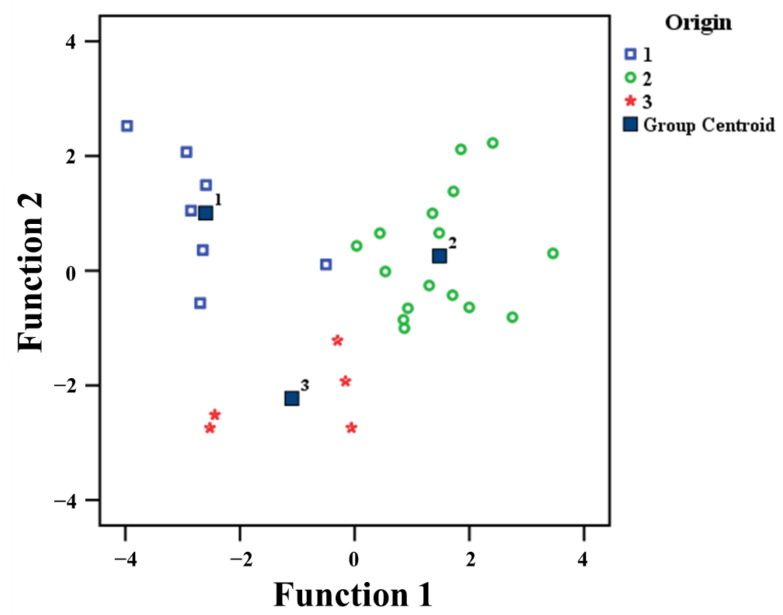
LDA of isotopic and elemental compositions using five markers for determining the geographical origins of THMR cultivated in three contiguous provinces in Thailand covering YP, RP, and SP. Empty squares, circles, and stars symbols refer to (1) YP, (2) RP, and (3) SP, respectively. Filled squares represent the group centroid of each province.

The isotopic (δ^18^O) and elemental composition (Mn, Rb, Co, and Mo) data combined with LDA were assigned to the groups of origins as YP (group 1), RP (group 2), and SP (group 3), respectively. Distribution patterns and group centroids of THMR in accordance with three contiguous provinces are also shown in Figure 5. The discriminant function 1 and function 2 were created from the markers. The canonical correlations were 0.883 (function 1), 0.754 (function 2), respectively. The first discriminant function explained 72.8% of total variance while the second discriminant function described 27.2% of the total variance. Function 1 was mainly correlated to Mn, Co, Rb, and Mo with standardized canonical discriminant function coefficients of 0.86, 0.51, −0.87, and 0.63, respectively. Function 2 was correlated to δ^18^O with a standardized canonical discriminant function coefficient of 0.84. LDA plotting by function 1 and function 2 is illustrated in Figure 5.

The LDA plot in Figure 5 illustrates the discrimination of THMR origins in three contiguous provinces. The origin of THMR in RP was successfully separated from other origins (YP and SP) using function 1. Function 2 discriminated clearly the origins of THMR cultivated between SP and other origins (YP and RP) which was not completely separated by function 1.

The cross-validation was also evaluated. An LDA model built with five variables (four elements and one stable isotope) provided 96.4% correct classification of the THMR in three origins and 92.9% of cross-validation as illustrated in Table 3. The combination of isotopic and elemental compositions with multivariate analysis was potentially effective in the origin traceability of THMR. These results implied that Mn, Rb, Co, Mo, and δ^18^O could be used as valuable markers for the discrimination of THMR cultivated from three contiguous provinces.

## 4. Conclusions

The isotopic and elemental analysis combined with powerful multivariate analysis is an alternative analytical tool in order to solve important problems regarding geographical origin traceability in worldwide agricultural products. Hence, this could be a promising method for classification of THMR. According to the THMR origins, registered as the European PGI, the discrimination of THMR using only five chemical markers was successfully accomplished with correct classification and cross-validation of 96.4% and 92.9%, respectively. Based on LDA, Mn, Rb, Co, Mo, and δ^18^O were valuable chemical markers to classify the geographical origins of THMR cultivated in three contiguous provinces in Thailand (*p* < 0.05). These results show that effective elemental/isotopic markers may provide insight for sustainable management and regulation of improper labeling regarding geographical origins of rice amongst Thailand and other countries. The database or the established system is also applicable to other countries and commodities.

## Figures and Tables

**Table 1:** Stable isotope and elements in THMR samples cultivated in three contiguous provinces.

Stable Isotope (‰)/Element (mg kg^−1^)	Province of Thai Hom Mali Rice Origin ^1^					
	YP (n =7)		RP (n = 16)		SP (n = 5)	
	Average	RSD ^2^	Average	RSD	Average	RSD
δ^18^O	25.48 a	0.018	25.42 a	0.03	24.20 b	0.018
Mn	17.71 c	0.26	12.74 d	0.23	12.82 cd	0.10
Co	0.085 e	0.89	0.039 f	2.03	0.033 ef	0.45
Rb	8.72 g	0.34	4.30 h	0.22	4.19 h	0.28
Mo	0.55 i	0.25	0.49 ij	0.32	0.32 j	0.34

^1^ The different letters indicate significant differences (*p* < 0.05) amongst different provinces. ^2^ Relative standard deviation.

**Table 2:** Classification of THMR according to the provinces of origins using linear discriminant analysis (LDA) based on each composition.

Classification	YP	RP	SP	Overall
LDA (%)	Elemental composition only			
	85.7	75.0	100.0	82.1
Cross-validated	71.4	75.0	80.0	75.0
LDA (%)	Combination of elemental and stable isotope compositions			
	85.7	100.0	100.0	96.4
Cross-validated	71.4	100.0	100.0	92.9

**Table 3:** LDA with correct classification and cross-validation of THMR in three contiguous provinces of origins using elemental and isotopic markers.

Origin				Prediction in 3 Provincial Origins		
			YP	RP	SP	Total
Classification	count	YP	6	1	0	7
		RP	0	16	0	16
		SP	0	0	5	5
	%	YP	85.7	14.3	0.0	100.0
		RP	0.0	100.0	0.0	100.0
		SP	0.0	0.0	100.0	100.0
Cross-validated	count	YP	5	1	1	7
		RP	0	16	0	16
		SP	0	0	5	5
	%	YP	71.4	14.3	14.3	100.0
		RP	0.0	100.0	0.0	100.0
		SP	0.0	0.0	100.0	100.0

## Data Availability

Not applicable.

## Supplementary Materials

The following are available online at https://www.mdpi.com/article/10.3390/foods10102349/s1, Table S1: Operating conditions of Agilent 7900 ICP-MS, Table S2: Method validation for the analysis of elemental composition in Thai Hom Mali rice using ICP-MS.
