# Supplementary material for: Tracing the Geographical Origin of Thai Hom Mali Rice in Three Contiguous Provinces of Thailand Using Stable Isotopic and Elemental Markers Combined with Multivariate Analysis
Source: Foods. 2021 Oct 1;10(10):2349. doi: 10.3390/foods10102349 (PMC8535565; doi:10.3390/foods10102349)
Supplement: Supplementary file 1 [file foods-10-02349-s001.zip › foods-1351598-supplementary.pdf]

foods MDPI

Supplementary Materials

Tracing the Geographical Origin of Thai Hom Mali Rice in Three Contiguous Provinces of Thailand Using Stable Isotopic and Elemental Markers Combined with Multivariate Analysis

Supalak Kongsri [1], Phitchan Sricharoen [1], Nunticha Limchoowong [2] and Chunyapuk Kukusamude [1,*]

[1] Nuclear Technology Research and Development Center (NTRDC), Thailand Institute of Nuclear Technology (Public Organization), 9/9 Moo 7, Saimoon, Ongkharak, Nakhon Nayok 26120, Thailand; supalak@tint.or.th (S.K.); phitchan@tint.or.th (P.S.)
[2] Department of Chemistry, Faculty of Science, Srinakharinwirot University, Sukhumvit 23, Wattana, Bangkok 10110, Thailand; nuntichoo@gmail.com
* Correspondence: chunyapuk@tint.or.th; Tel.: +66-085-484-6782 (ext. 1803)

Table S1. Operating conditions of Agilent 7900 ICP-MS.

Operating Parameters	Condition
RF power	1550 W
RF matching	1.70 V
Sampling depth	8.0 mm
Carrier gas flow rate	0.80 L/min
Nebulizer speed	0.10 rps
Chamber temperature	2 °C
Nebulizer	Mira Mist
Spray chamber	Scott type
Replicates	3
Sample and skimmer cones	Ni

Table S2. Method validation for the analysis of elemental composition in Thai Hom Mali rice using ICP-MS.

Element	R^2	Certified Value [a] (mg kg^{-1})	Measured Value (mg kg^{-1})	Recovery (%)	LOD (µg kg^{-1})	LOQ (µg kg^{-1})	Precision (% RSD)			
							Repeatability [b] (%RSD_r)		Intermediate [c] (%RSD_I)	
							Level 1	Level 2	Level 1	Level 2
Mn	0.9990	19.2	18.94	98.64	1.38	2.00	1.18	0.89	6.08	7.68
Co	1.0000	0.0177	0.0179	101.36	0.29	0.83	3.10	1.20	8.68	7.13
Rb	0.9998	6.198	6.234	100.58	11.8	12.4	1.09	0.56	5.60	3.62
Mo	0.9999	1.451	1.435	98.87	0.50	0.88	1.55	1.67	3.53	4.46

[a] The reference material is NIST 1568b. [b] 2 concentration levels × 3 replicates [c] 2 concentration levels × 3 replicates × 4 days.
